# Supplementary material for: Multimodal retinal imaging by visible light optical coherence tomography and phosphorescence lifetime ophthalmoscopy in the mouse eye
Source: Neurophotonics. 2025 Sep 27;12(3):035015. doi: 10.1117/1.NPh.12.3.035015 (PMC12476265; doi:10.1117/1.NPh.12.3.035015)
Supplement: Supplementary file 1 [file NPh_012_035015_SD001.pdf]

Supplementary Material for "Multimodal retinal imaging by visible light optical coherence tomography and phosphorescence lifetime ophthalmoscopy in the mouse eye"

Section S1: Simultaneous Imaging Signal Protocol

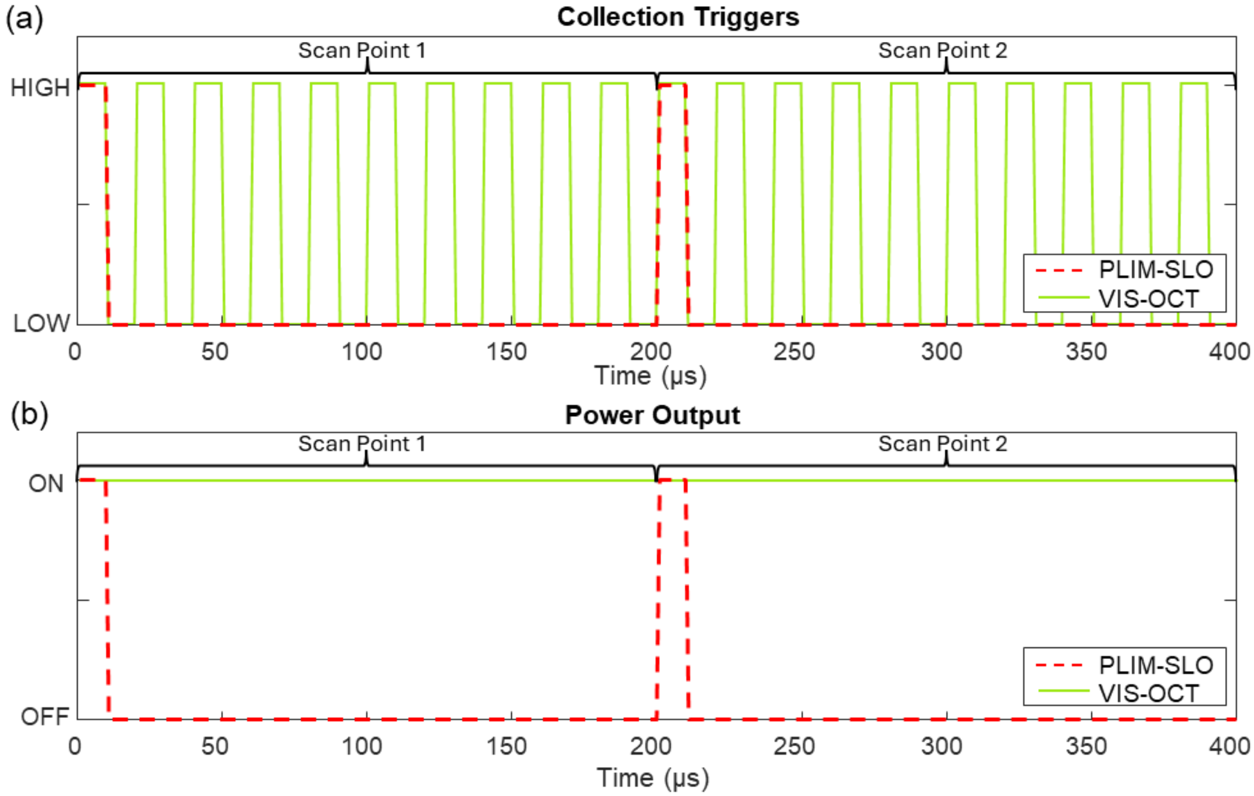

Fig. S1. Protocol for simultaneous data acquisition. Two collection points during the raster scan are shown. (a) The PLIM detector is triggered once for the full 200 μs collection at each point, while the VIS-OCT channel is triggered ten times for a 50 kHz imaging rate. (b) The VIS-OCT power remains constant during each 200 μs dwell time, while the PLIM pulse is delivered for 10 μs at the start of each collection.

Section S2: ANSI Safety Calculation for Simultaneous Imaging

Simultaneous imaging provides the highest total light exposure risk, so ocular safety was assessed for this protocol. During simultaneous imaging, VIS-OCT had 0.3 mW continuous power, while PLIM-SLO had one 5 mW pulse for 10 μs at the start of each 200 μs scanning location (treated as a point source on the retina). The VIS-OCT supercontinuum light source had a pulse rate of 80 MHz, and given the pulses had equal power and high repetition frequency, ANSI Rule 2 for multi-pulse exposures provided the more restrictive maximum permissible exposure (MPE) than Rule 1 and was used[51]. For the dwell time of 200 μs, the MPE was $3.03\text{x}10^{-6}$ J cm^{-2} and gave a

max VIS-OCT average power of 5.83 mW. For PLIM, the MPE for the 10 us pulse was calculated as 3.20×10^{-7} J cm^{-2}, which translated to a max pulse power of 12.32 mW.

For combined imaging, the powers used were normalized to the MPE powers for each channel and combined[52]. During simultaneous imaging the 0.3/5.83 VIS-OCT and 5/12.32 PLIM-SLO ratios were summed to 0.46, which fell within the safe level below one. To consider the effects of multiple collections over time, the most dramatic cumulative scenario would include a continuously running simultaneous protocol without eye movement. Each point location would receive the 10 μs, 5 mW PLIM-SLO pulse and the 200 μs burst of 0.3 mW VIS light every 13.1 s. Each individual pulse was already deemed safe, so Rule 2 for average power was tested. For total exposure durations lasting over 10s, the max permissible power would be 0.385 mW for each channel[51]. The time required for the scanning beam to return to its original position brought the average power very low for each channel (3.82×10^{-6} mW from PLIM-SLO and 4.58×10^{-6} mW from VIS-OCT). In practical imaging conditions, additional time always passes between collections, and the beam is never static on the retina. Total light exposure over time is further minimized by using a reduced-power SLO preview protocol during alignment, during which the VIS channel is blocked.

Section S3: Derivation of Decay Equation with Continuous Wave (CW) Background Excitation

The rate of change in the number of the excited state triplet (T_1) species (n^*) following the excitation pulse in the presence of the background CW illumination is described as:

$$\frac{dn^*}{dt} = -n^* \times k_{Ox2P} + n_0 \times k_{OCT}, \qquad (S1)$$

Where t is time, n_0 is the number of the ground state (S_0) species, k_{Ox2P} is the rate constant for the T_1 depopulation due to radiative (phosphorescence) and non-radiative processes (including quenching by oxygen), k_{OCT} is the absorption rate constant due to the CW excitation by the OCT laser, n^* is the number of probe molecules in the excited state. We consider that n is the total number of probe molecules in the volume, and it is constant, so that:

$$n = n^* + n_0, \qquad (S2)$$

We ignore the stimulated emission term, since the energy of the triplet state is significantly lower than the excitation energy, and the respective transition ($T_1 \rightarrow S_0$) is spin-forbidden. We also consider the transition from the initially populated excited state (S_1) to T_1 (intersystem crossing) instantaneous relative to the rate constants k_{Ox2P} and k_{OCT}.

Substituting Eqn. S2 into Eqn. S1, gives a nonhomogeneous linear differential equation:

$$\frac{dn^*}{dt} + (k_{Ox2P} + k_{OCT})n^* = nk_{OCT}, \tag{S3}$$

The complementary equation is separable, so the complementary solution $n_c^*(t)$ is:

$$\frac{dn^*}{dt} + (k_{Ox2P} + k_{OCT})n^* = 0,$$

$$\int \frac{1}{n^*} dn^* = \int -(k_{Ox2P} + k_{OCT})\, dt,$$

$$n_c^*(t) = Ae^{-(k_{Ox2P} + k_{OCT})t}, \tag{S4}$$

For the particular solution, the constant $n_p^*(t)$ = B is used as the guess for the solution:

$$0 + (k_{Ox2P} + k_{OCT})B = nk_{OCT},$$

$$n_p^*(t) = B = \frac{nk_{OCT}}{(k_{Ox2P} + k_{OCT})}, \tag{S5}$$

Therefore, the solution is:

$$n^*(t) = n_c^*(t) + n_p^*(t) = Ae^{-(k_{Ox2P} + k_{OCT})t} + \frac{nk_{OCT}}{(k_{Ox2P} + k_{OCT})}, \tag{S6}$$

Using the initial value at t=0, $n^*(0) = n^*_{t=0}$ to solve for A gives the final formula:

$$n^*(t) = \left(n_{t=0}^* - \frac{nk_{OCT}}{(k_{Ox2P} + k_{OCT})}\right) e^{-(k_{Ox2P} + k_{OCT})t} + \frac{nk_{OCT}}{(k_{Ox2P} + k_{OCT})}, \tag{S7}$$

Therefore, when performing simultaneous imaging, the rate constant that is calculated by the fitting, k_{Simul}, is equal to the sum of k_{Ox2P} and k_{OCT}, and the phosphorescence decay is superimposed on a constant background:

$$I_{Simul}(t) = I_C e^{-(k_{Simul})t} + I_{SS}, \tag{S8}$$

$$k_{Simul} = k_{Ox2P} + k_{OCT}, \tag{S9}$$

The constant I_C is the intensity of the phosphorescence signal excited by the pulse at the start of the decay, while the constant I_{SS} is the intensity of phosphorescence due to the CW VIS-OCT illumination.

The excitation rate constant k_{OCT} can be estimated by considering:

$$k_{OCT} = \sigma \times \Phi, \tag{S10}$$

where σ is the molecular excitation cross-section at the wavelength of the OCT laser and Φ is the photon flux through the excitation volume. The molecular excitation cross-

section is proportional to the molar extinction coefficient ε, which for Ox2P is on the order of 10^2-10^3 $M^{-1}cm^{-1}$ near 500 nm. The cross-section σ is found as:

$$\sigma = \frac{\ln(10) \times 1000 \times \varepsilon}{N_a}, \tag{S11}$$

where N_a is the Avogadro number. The photon flux is defined as:

$$\Phi = \frac{P \times \lambda}{h \times c \times S}, \tag{S12}$$

where P is the OCT laser power at the focus (i.e. attenuated due to tissue absorption and scattering), λ is the excitation wavelength, h is the Planck constant, c is the speed of light in the medium, and S is the beam cross-section at the waist.

While the exact value for P in the focal volume is difficult to measure/estimate, attenuation of the power, compared to the measured incident power under the objective, by 5-10 times, due to the absorption/scattering, results in the excitation rate constant values constituting 10-20% of the native decay constant k_{Ox2P} at physiological oxygen pressure of 30 mmHg. These estimates are in good agreement with experimentally measured correction values k_{corr} (see main text).

Section S4: Sample of Average pO_2 of each Vessel Type over Depth

The tunable lens was used to section through the retina from the surface vessels to the deep capillary plexus and estimate the depth position. The average arterial, capillary, and venous values were plotted over depth (Fig. S2 a), revealing a decrease in capillary pO_2 with depth. The trend lines were calculated by linear fitting and shown with each vessel type. The difference between the arteries and capillaries as well as the capillaries and veins were also included to show the capillary values relative to the major vessels (Fig. S2 b). As depth increased, the capillary values started closer to the arterial values, then became closer to the venous values. Sample planes of the intensity and average pO_2 are included to show vessel grouping for calculation. The sample planes correspond to three points plotted in Fig. S2 a-b, starting at the retina surface: point 1 (Fig. S2 c), point 5 (Fig. S2 d), and point 8 (Fig. S2 e). Arteriolar microvessel branches are more apparent towards the surface, while feeding venular branches become focused towards the deeper retina.

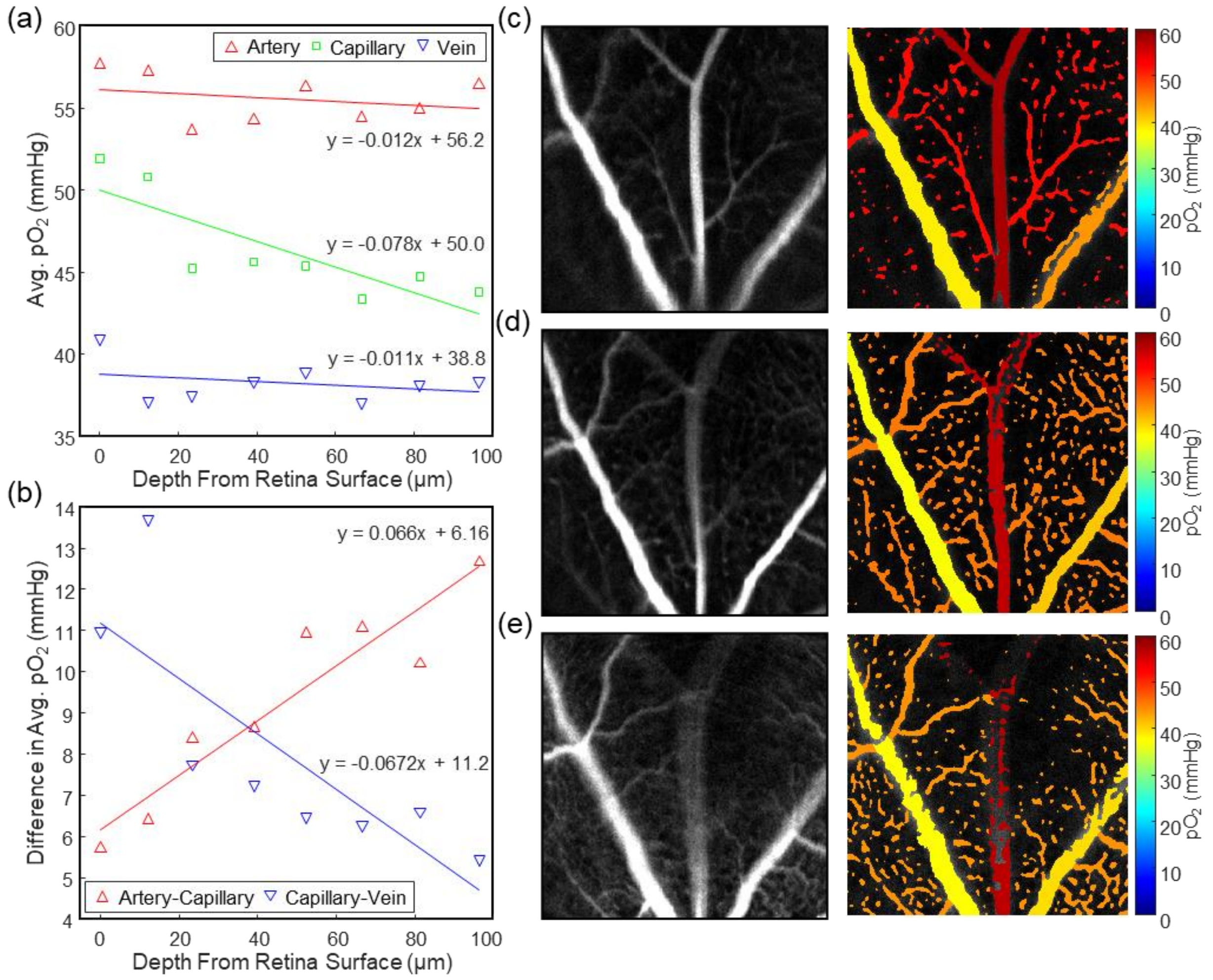

Fig. S2. Plots of pO₂ over increasing depth. (a) The average arterial, venous, and capillary pO_2 values are plotted across increasing depth in the retina. The average capillary pO_2 was shown to decrease as the depth into the retina increased. (b) The difference between arteries and capillaries increased as depth increased, while the difference between capillaries and veins decreased over depth. Intensity and average pO_2 maps show vessel distributions and groupings corresponding to the (c) first, (d) fifth, and (e) eighth plotted points.

Section S5: Estimation of A-V Difference and Oxygen Extraction Fraction using Oxygen Saturation and Content

To account for the nonlinear binding of oxygen with hemoglobin (Hb), additional oxygen metrics were estimated for comparison to oxygen metabolism values in the literature.

First, oxygen saturation was calculated from the measured pO_2 using the Hill equation (in both arteries and veins for consistency) with the coefficients discussed in the main text: n = 2.59 and p50 = 40.2 mmHg[58]. Oxygen content (cO_2), which measures the overall concentration of oxygen in the blood (mL O_2/dL blood), was estimated from the

pO$_2$ and calculated sO$_2$ using a Hb carrying capacity of 1.34 mL O$_2$/g Hb and oxygen solubility of 0.003 mL O$_2$/dL blood/mmHg:

$$cO_2 = 1.34\left(\frac{mL\ O_2}{g\ Hb}\right) \times [Hb]\left(\frac{g\ Hb}{dL}\right) \times sO_2 + pO_2(mmHg) \times 0.003\left(\frac{mL\ O_2}{mmHg\ dL}\right) \quad (S13)$$

A hemoglobin concentration of 15.2 g Hb/dL blood was assumed for healthy C57BL/6 male mice[59]. The arteriovenous (A-V) difference was calculated from the average arterial and venous sO$_2$ and cO$_2$ values during each inhaled oxygen state collection. A least-squares linear fit was applied to the A-V differences plotted against the arterial values (Fig. S3 a-b).

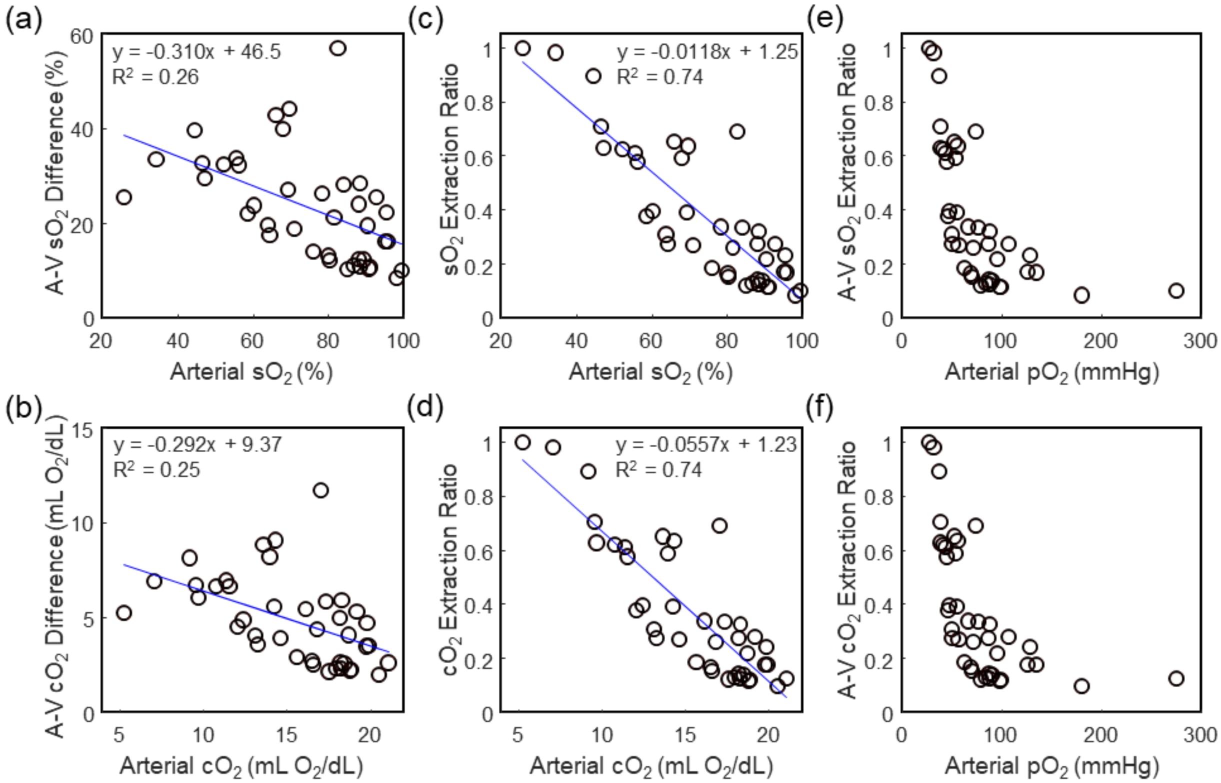

Fig. S3. A-V differences and oxygen extraction ratios. (a) The A-V sO$_2$ difference had a decreasing slope as arterial saturation increased. (b) Similarly, the A-V cO$_2$ difference had a decreasing slope as arterial content increased. The oxygen extraction ratio calculated from the (c) sO$_2$ and (d) cO$_2$ also showed decreasing slopes when arterial sO$_2$ and cO$_2$ increased, respectively. (e-f) When the extraction ratios were plotted against the pO$_2$, the trend no longer appeared linear but still revealed lower extraction ratios with higher levels of dissolved oxygen.

While the total oxygen extraction metric requires blood flow information, we can use a relative metric called the oxygen extraction fraction, also known as the oxygen extraction ratio. This ratio has been previously calculated in the literature from the A-V difference over arterial value of sO$_2$[25] or cO$_2$[60] and has been shown to decrease as oxygen increases in rats[25,60]. Both methods were calculated and plotted against the

arterial sO_2 or cO_2 (Fig. S3 c-d) and the arterial pO_2 (Fig. S3 e-f). A linear fit was applied to the oxygen extraction ratios plotted against the arterial values. When the ratio is plotted against the pO_2 to allow better discernment of hyperoxia vs normoxia, the trend no longer appears linear, so a linear fit was not applied.
